# Internet‐Based Acceptance and Commitment Therapy With Interoceptive Exposure for Panic Disorder: A Randomized Controlled Trial and Working Alliance Analysis

**DOI:** 10.1111/sjop.70045

**Published:** 2025-11-28

**Authors:** Lisa Bäckman, Sandra Weineland, Kristofer Vernmark, Ella Radvogin, Pär Bjälkebring, Esther Enbuske, Ida Hermansson, Nina Johansson, Nathalie Petersen, Timo Hursti

**Affiliations:** ^1^ Department of Psychology University of Gothenburg Gothenburg Sweden; ^2^ Department of Research, Education and Innovation Region Västra Götaland, Södra Älvsborg Hospital Borås Sweden; ^3^ General Practice/Family Medicine, School of Public Health and Community Medicine Institute of Medicine, Sahlgrenska Academy, University of Gothenburg Gothenburg Sweden; ^4^ Research, Education, Development & Innovation, Primary Health Care, Region Västra Götaland Sweden; ^5^ Department of Behavioural Sciences and Learning (IBL) Linköping University Linköping Sweden; ^6^ Psykologpartners Stockholm Stockholm Sweden; ^7^ Department of Psychology University of Uppsala Uppsala Sweden

**Keywords:** acceptance and commitment therapy, agoraphobia, cognitive behavioral therapy, internet‐based, interoceptive exposure, panic disorder, working alliance

## Abstract

This study's primary aim was to evaluate the efficacy of an internet‐based Acceptance and Commitment Therapy (IACT) program modified to include interoceptive exposure for treating panic disorder with or without concurrent agoraphobia. Its secondary aim was to examine whether therapist‐ and client‐rated working alliances were related to treatment outcomes. This randomized controlled trial included 79 participants, assigned to either a treatment group (*n* = 40) or a waitlist control group (*n* = 39) over 10 weeks. The study investigated the effects on panic disorder and quality of life, as well as the relationship between working alliances (rated by therapists and clients) and treatment outcomes. At post‐treatment, there was a significant between‐group treatment effect on panic disorder symptoms, with an observed effect size of *d* = 0.92. The model‐predicted effect size based on the multilevel model was *d*_GMA‐raw_ = 0.86. Furthermore, 43% of participants no longer met the diagnostic criteria. Participants with concurrent agoraphobia exhibited higher initial panic symptom scores and were less likely to be diagnosis‐free post‐treatment. However, they still experienced significant and large treatment effects, with an observed effect size *d* = 1.22 and *d*_GMA‐raw_ = 0,99. There was no significant between‐group difference in quality‐of‐life measurements. The therapist‐rated working alliance was associated with treatment outcome, but no significant relationship was found for the client‐rated alliance. Overall, the study suggests that interoceptive exposure‐modified IACT is an effective treatment for panic disorder and shows promise for patients with concurrent agoraphobia.

**Summary**

- IACT with interoceptive exposure significantly reduced panic symptoms compared to waitlist, including among participants with concurrent agoraphobia.
- Quality of life improved within treatment but not significantly compared to waitlist.
- Increases in therapist‐rated, but not client‐rated, working alliance was associated with better outcomes.
- Integrating interoceptive exposure within ACT appears to be a feasible and effective approach for panic disorder.

## 1 Introduction

Panic Disorder (PD) significantly impacts individuals' lives, with a cross‐national lifetime prevalence estimated at 1.7% globally and 2.2% in Sweden (Carlbring et al. 2002; de Jonge et al. 2016). Individuals with PD often find that they react to symptoms as if they are in actual physical danger, resulting in increased healthcare usage and significantly higher costs compared to other psychiatric conditions (Batelaan et al. 2007; Hohls et al. 2019). With concurrent agoraphobia, PD is linked to higher rates of substance use disorder, social anxiety, specific phobia, generalized anxiety disorder, and an elevated risk of suicide (Grant et al. 2006; Inoue et al. 2016). PD and agoraphobia require accessible evidence‐based care, making it essential to explore diverse treatments, with internet‐based interventions highlighted as a potentially central approach for managing these conditions (Domhardt et al. 2020). The aim of this study is to evaluate the efficacy of an internet‐based Acceptance and Commitment Therapy (IACT) program, modified to include interoceptive exposure, for treating panic disorder with or without concurrent agoraphobia.

Cognitive Behavioral Therapy (CBT) is a well‐established treatment for PD (Pompoli et al. 2018; Rabasco et al. 2022). However, since CBT is not effective for everyone with PD or agoraphobia, this highlights the need to investigate alternative treatment approaches (Gloster et al. 2015; Meuret et al. 2012; Socialstyrelsen 2017). A recent umbrella review highlights Acceptance and Commitment Therapy (ACT) as a treatment for PD (Rabasco et al. 2022). There are studies demonstrating ACT's effectiveness, including for PD and agoraphobia; however, they also highlight the need for further research (A‐Tjak et al. 2015; Gloster et al. 2020).

Both CBT and ACT address inner experiences such as thoughts, emotions, and sensations, but they differ in their approach. CBT focuses on identifying and modifying the content of an individual's experiences. In contrast, ACT emphasizes changing the individual's relationship with these experiences rather than altering their content. ACT promotes psychological flexibility through acceptance and mindfulness practices. In this way they can replace avoidance behaviors with actions aligned with their values, even when faced with discomfort (Hayes et al. 2013).

In ACT, PD is understood to be maintained by experiential avoidance, where efforts to eliminate panic symptoms hinder individuals from pursuing meaningful personal goals (López and Salas 2009). Patients are encouraged to accept inner sensations, observing them mindfully rather than approaching them as threats, thereby lessening avoidance or suppression efforts (Eifert et al. 2009; Levitt et al. 2004). In CBT, systematic interoceptive exposure is emphasized as central to the effectiveness of PD treatment and can lead to habituation and/or inhibitory learning (Craske and Barlow 2014). While habituation aims to reduce fear, inhibitory learning focuses on managing fear by creating new, adaptive associations (Craske and Barlow 2014). This makes inhibitory learning more sustainable, allowing patients to function even if fear persists. ACT's observational approach can enhance learning during exposure, and a pilot study combining ACT and interoceptive exposure has shown positive outcomes (Meuret et al. 2012). The possibility of increased observational ability and acceptance in the presence of feared stimuli aligns with Craske et al. (2008), who suggest tolerating exposure is more important than simply reducing fear.

In Sweden, as in many other countries, the demand for evidence‐based psychological treatment exceeds the capacity of primary and psychiatric care (McManus et al. 2014; Socialstyrelsen 2021). One way to reach a larger number of patients is through internet‐based interventions. Therapist‐guided internet‐based CBT (ICBT) has demonstrated effects comparable to traditional face‐to‐face CBT, including for PD (Hedman‐Lagerlöf et al. 2023; Pauley et al. 2023). Preliminary evidence further supports the efficacy of internet‐based ACT (IACT; Han and Kim 2022; Thompson et al. 2021). However, studies assessing the impact of IACT on PD or agoraphobia remain inconclusive (Gloster et al. 2020; Kelson et al. 2019).

In January 2024 there were 5986 ongoing ICBT programs (including IACT) for anxiety disorders, depression, and insomnia, provided through the Swedish national platform for internet‐based treatment (Vernmark et al. 2024). The most frequently used intervention is the IACT program *Anxiety Help (Ångesthjälpen)*, with 1168 ongoing programs as of January 2025 and a total of 18,706 programs started since 2016 (Inera, n.d.). In a randomized controlled trial, this transdiagnostic treatment program demonstrated a medium between‐group effect for social phobia but no significant effect for PD (Ivanova et al. 2016). However, the study was underpowered for the PD subsample analysis and the within‐group effect size for the treatment group indicated greater improvements than the waitlist control. Since this evaluation, the Anxiety Help program has been modified to include CBT‐based interoceptive exposure elements.

Anxiety Help is a guided internet‐ based intervention that involves therapist support, often through asynchronous written feedback. Therapists guide and motivate clients by providing support, personalizing content, and tracking progress. Research indicates that guided online treatments are generally more effective (Baumeister et al. 2014; González‐Robles et al. 2024), However, studies analyzing which specific behaviors are associated with treatment effects and adherence are still scarce (Holländare et al. 2015).

Working alliance is a pan‐theoretical concept that has been found to correlate significantly with treatment outcomes in various forms of psychotherapy. As guidance is a critical factor in internet‐based treatments, the role of working alliance in this format has gained the interest of researchers (Andersson et al. 2012; Vernmark et al. 2019). In a meta‐analysis by Flückiger et al. (2018), the overall correlation between alliance and treatment outcome was comparable in face‐to‐face treatments (*r* = 0.28) and internet‐based treatments (*r* = 0.28). The alliance‐outcome effect sizes in internet‐based treatments were in this meta‐analysis more homogeneous, demonstrating less variability compared to face‐to‐face studies. This indicates a more stable association between patients' working alliance and outcomes in online settings. A similar correlation (*r* = 0.22) was observed when the therapist‐rated alliance was assessed, albeit here the data predominantly originated from face‐to‐face treatment (Flückiger et al. 2018). According to Flückiger (2022), no clear pattern of systematic differences exists between alliance raters, including patients, therapists, or other observers.

However, there is still limited research on how the therapist assesses the working alliance in internet‐based treatment (Berger 2017). Vernmark et al. (2019) found that therapist‐rated alliance correlated more strongly with outcomes in a blended CBT treatment than patient‐rated alliance. To better understand the potential role of the working alliance in internet‐based treatment, additional investigations, including repeated assessments for exploring temporality, are still needed (Cuijpers et al. 2019; Flückiger et al. 2020). Thus, the secondary aim of this study is to investigate if therapist‐ and patient‐rated working alliance is related to treatment outcomes.

Research on IACT, alone and combined with interoceptive exposure, remains limited. This study aimed to evaluate the efficacy of an internet‐based Acceptance and Commitment Therapy (IACT) program modified to include interoceptive exposure for treating panic disorder with or without concurrent agoraphobia. It also examined whether agoraphobia impacts the effects of treatment and whether weekly therapist and participant ratings of the working alliance, as well as the overall alliance measured across all weeks as a process, predict treatment outcomes.

## 2 Materials and Methods

### 2.1 Participants

This study included 79 participants with PD (with or without agoraphobia). Participants were included if they scored eight or higher on the Panic Disorder Severity Scale–Self‐Rated (PDSS‐SR) and met the criteria for PD according to version 7.0.0 of the Mini‐International Neuropsychiatric Interview (MINI). They needed to be proficient in spoken and written Swedish, have access to a computer, tablet, or smartphone with an internet connection, and be between 18 and 65 years old.

Exclusion criteria following the online screening included severe alcohol dependence and suicidal ideation. Additional exclusion criteria, assessed during the telephone interview, encompassed individuals with complex clinical problems that could significantly hinder participation in the treatment. Participants meeting the MINI criteria for severe depression, bipolar disorder, PTSD, substance use disorders, psychotic symptoms, eating disorders, antisocial personality disorder, or an elevated risk of suicide were excluded. Furthermore, individuals with a primary diagnosis of depression, social anxiety disorder, obsessive‐compulsive disorder (OCD), or generalized anxiety disorder, where PD was deemed secondary, were excluded from the study.

**FIGURE 1:**
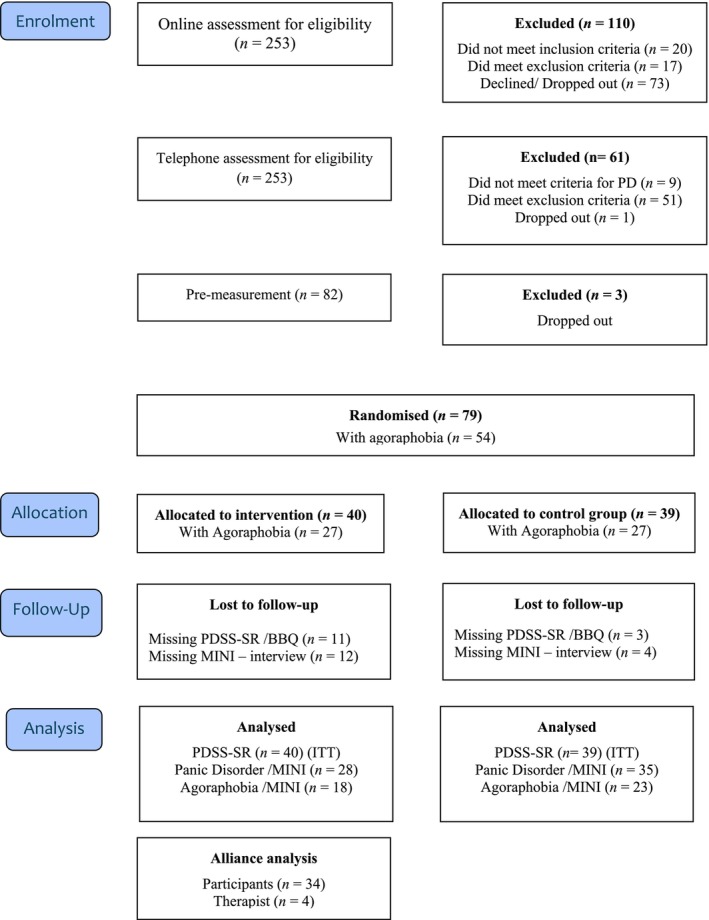
CONSORT flowchart of participants.

Of the 253 participants screened online, 143 qualified for an in‐depth telephone assessment, and 82 were included and agreed to participate. Three participants dropped out before randomization, resulting in 79 participants (see CONSORT flowchart in Figure 1). Among these, 54 met the criteria for agoraphobia. The average age of the participants was 34 (*M* = 34, SD = 10; range: 18–58), and 90% were women. Additional demographic data are presented in Table 1.

**TABLE 1:** Demographic characteristics of treatment group (*n* = 40) and waitlist control group (*n* = 39).

Variable	Treatment group (*n* = 40)	Waitlist control (*n* = 39)
Age, mean (SD)	34.5 (9.9)	33.1 (10.4)
Gender		
Female	37 (92.5%)	34 (87.2%)
Male	3 (7.5%)	4 (10.3%)
Non‐binary	0 (0)	1 (2.5%)
Occupation		
Working/student	31 (77.5%)	26 (66.7%)
Unemployed	1 (2.5%)	6 (15.4%)
Sick leave	8 (20%)	7 (17.9%)
Other	0 (0)	0 (0)
Education		
Compulsory education	3 (7.5%)	3 (7.7%)
Upper secondary education	17 (42.5%)	23 (59%)
3 years of tertiary education	9 (22.5%)	7 (17.9%)
> 3 years of tertiary education	11 (27.5%)	6 (15.4%)
Residence		
City (≥ 200,000 inhabitants)	5 (12.5%)	11 (28.2%)
Small city (40000–200,000 inhabitants)	17 (42.5%)	10 (25.6%)
Small town (15000–40,000 inhabitants)	7 (17.5%)	7 (17.9%)
Village (< 15,000 inhabitants)	11 (27.5%)	11 (28.2%)
Marital status		
Married/partnered	17 (42.5%)	29 (74.4%)
Divorced	4 (10%)	2 (5.1%)
Single	19 (47.5%)	8 (20.5%)
Current medication		
Regular	10 (25%)	13 (33.3%)
If needed	3 (7.5%)	9 (23%)
Both regular and if needed	3 (7.5%)	2 (5.1%)
None	24 (60%)	15 (38.4%)
Previous psychological treatment		
Yes	21 (52.5%)	30 (76.9%)
No	19 (47.5%)	9 (23%)

### 2.2 Procedures

The Regional Ethical Review Board approved the study in Uppsala, Sweden (Dnr: 2018/451). Although the study was not pre‐registered, the aims of the RCT, the sample size, and possible comparisons were part of the design that received ethical approval before data collection began.

Participants were recruited through information posters distributed in various public locations and selected Facebook groups, as well as through paid Facebook advertising. These advertisements included a brief description of the study and referred to the project's website, where participants could find further information about the research and sign up for participation.

Following informed consent, participants underwent online screening. Qualified participants took part in a diagnostic phone interview with one of the study's four therapists using version 7.0.0 of MINI. The interviewer could consult a licensed psychologist for guidance on inclusion or exclusion. Excluded participants were contacted via email. If suicide risk was the reason for exclusion, they were contacted by telephone. Excluded participants were advised to seek support through primary care centres, psychiatric services, the National Helpline or the nearest psychiatric emergency department for acute problems.

Due to a two‐month gap between the initial screening and the start of treatment, a pre‐assessment was conducted 1 week prior to treatment using the primary outcome instrument. At this point, fourteen individuals exhibited symptoms below the cut‐off. These participants were included in the study for ethical reasons, as they had met the criteria for a PD diagnosis during recruitment. This inclusion was based on the assumption that symptoms could vary more than the diagnosis over time. Furthermore, including these participants in the treatment was deemed to pose no risk or harm to them.

Eligible participants were randomly assigned to either the treatment or control (waitlist) group using an online random generator (www.randomizer.org). A blinded researcher conducted the allocation, ensuring an equal distribution of participants with agoraphobia across both groups. Therapists were assigned patients they had not previously interviewed, and each therapist managed nine to eleven patients. Participants were not compensated for their involvement in the study.

### 2.3 Outcome Measures

The effects of the intervention were evaluated using the Swedish version of the Panic Disorder Severity Scale (PDSS‐SR) as the primary outcome measure and the Brunnsviken Brief Quality of Life Scale (BBQ) as the secondary outcome measure (Lindner et al. 2016; Svensson et al. 2019). The PDSS‐SR assesses the frequency of panic attacks, discomfort during attacks, anxiety, agoraphobic fear, avoidance, fear of physical sensations, and the impact on work and social functioning. The PDSS‐SR comprises seven items on a 5‐point Likert scale (0–4), demonstrating good internal consistency (Cronbach's α = 0.80–0.93; depending on the measurement point). It also exhibits adequate test–retest reliability (*r* = 0.70) and high sensitivity in evaluating PD treatments (Svensson et al. 2019). BBQ measures quality of life across six areas and includes 12 items on a 5‐point Likert scale (0–4). BBQ demonstrates strong test–retest reliability (*r* = 0.82) and validity, though its internal consistency is moderate (Cronbach's *α* = 0.76). It is recommended for use in clinical and research settings (Lindner et al. 2016).

The Working Alliance Inventory‐Short Revised (WAI‐SR) assessment, a 12‐item self‐assessment tool based on Bordin's (1979) theory, was used to evaluate the working alliance (Hatcher and Gillaspy 2006). WAI‐SR measures three main components: bond, shared tasks, and therapeutic goals. It is commonly used in outcome studies (Mallinckrodt and Tekie 2016; Martin et al. 2000). WAI‐SR employs a 5‐point Likert scale and is available in both client (WAI‐SR‐C) and therapist (WAI‐SR‐T) versions. WAI‐SR‐C demonstrates good internal consistency (Cronbach's *α* > 0.80) for subscales and total score (Cronbach's *α* > 0.90; Munder et al. 2010). WAI‐SR‐T exhibits satisfactory psychometric properties regarding factor structure, item information properties, and measurement invariance (Hatcher et al. 2020). This study used a composite score, averaging individual item scores (1–5), where higher values indicate a stronger alliance. The WAI‐SR‐T consist of 10 items, leading to a lower total score than the 12‐item WAI‐SR‐C.

### 2.4 Screening Measurements

The online screening instrument included PDSS‐SR, the Alcohol Use Disorders Identification Test (AUDIT; Bergman and Källmén 2002) to assess hazardous or harmful alcohol consumption with a cutoff score of 19 and the Patient Health Questionnaire‐9 (PHQ‐9; Titov et al. 2011) to identify suicidal ideation, using a cutoff score of three on Item 9.

### 2.5 Diagnostic Interview Assessment

MINI 7.0.0 developed by Sheehan et al. (1998) is a semi‐structured diagnostic interview guide incorporating clinical judgment to ensure accurate diagnosis. MINI was used to assess criteria for PD and agoraphobia, confirm PD as the primary diagnosis, and screen for exclusionary diagnoses.

### 2.6 Assessment Schedule

One week before treatment began, an updated baseline measurement was conducted, including participants' reports on demographic data and ongoing psychopharmacological medication. Throughout the treatment, participants completed weekly PDSS‐SR registrations, and both the participant and therapist answered WAI‐SR weekly (10 times). Additionally, participants completed post‐treatment measurements for BBQ and PDSS‐SR. The MINI 7.0.0 was conducted via telephone post‐treatment to evaluate whether participants met the criteria for PD and agoraphobia.

### 2.7 Intervention

Anxiety Help is a therapist‐guided, internet‐based ACT treatment (see Table 2). It is designed to help people with various types of anxiety, such as social phobia, panic disorder, agoraphobia, generalized anxiety, health anxiety, and/or performance anxiety. Developed by Psykologpartners, a private psychology company in Sweden, the program was launched in 2011. It is commercially available and has been implemented all over Sweden through collaborations with Swedish public healthcare regions.

**TABLE 2:** Modules in the treatment program.

Module	Title of module	Core treatment component	Content, focus and example of exercises
1	A first step	Psychoeducation and preparation for treatment	Plan specific dates, times, and places to work with the program Observe and register “monsters” (e.g., anxiety) over the coming week New addition: There is an optional link to information about various anxiety diagnoses, including PD
2	Functional analysis	Get acquainted with functional analysis, including how short‐ and long‐term consequences can conflict	Conduct at least three functional analyses on anxiety‐provoking situations Reflect on how your life would change if your anxiety disappeared
3	Goals and obstacles	Define personal values to find motivation for long‐term strategies rather than avoidance and control	Define personal values and identify the most important areas Develop strategies to address obstacles in different areas of life Practise functional analyses and work on living according to your personal values
4	Meet your monsters in a new way	Acceptance as an alternative to control and avoidance Move in a valued direction despite discomfort	Analyze what can be labeled “pure” and “dirty” discomfort Make active choices for moving in a valued direction while making room for any discomfort that may arise New addition: *Exposure*: Guidance on using exposure techniques, including the consideration of safety behaviors and the possibility of heightened anxiety during the initial stages. The therapeutic approach also focuses on supporting the tolerance of experiences and reflecting on how these experiences align or differ from their expectations. The aim is to enhance self‐awareness and strengthen the ability to engage with challenging situations Inner exposure exercises that focus on both interoceptive and imaginative exposure
5	Thoughts and feelings	Thoughts and feelings do not have to control your actions	Engage in experiential exercises, such as the “Observer exercise” Write down life rules
6	Mindfulness	Use mindfulness to connect with what is important in life	Mindfulness exercises Practice mindfulness for 15 min on three occasions in the coming week
7	Valued direction	Work with functional analyses, values, mindfulness, and acceptance through concrete actions in a valued direction	Choose two life areas to focus on and take meaningful steps in a valued direction, while practising acceptance
8	Actions that work	Evaluation and summary of the program	Review learnings and plan for potential setbacks

Based on the findings from the Anxiety Help trial, Ivanova et al. (2016) recommended incorporating more detailed instructions and examples to enhance patient engagement with the transdiagnostic approach. Research has also shown that repeated interoceptive exposure (Arch and Craske 2008; Craske and Barlow 2008, 2014) and integrating mindfulness with exposure therapy can reduce avoidance and improve outcomes (Arch et al. 2012). Interoceptive exposure was therefore incorporated into one of the modules (Module 4, see Table 2 and Table S1). The remaining modules consistently adhered to the ACT rationale. This involved emphasizing acceptance and recognizing and understanding what can and cannot be controlled, embracing difficult emotions, and promoting effective behaviors rather than avoidance. In the treatment, the combination of acceptance and interoceptive exposure is demonstrated by learning to manage anxiety through various, intentional exposures and how discomfort faced during exposure can be managed through acceptance and engagement in meaningful activities. Through these new experiences, patients can learn that they can cope with discomfort and that anxiety is not harmful. An overview of the program and the addition of interoceptive exposure can be found in Table 2. Further information about the treatment program can be found in (Table S1; Table S2; Figure S1).

The program consists of eight online modules, intended to be used weekly for 10 weeks. It is delivered via text, video clips, and audio files and contains information, strategies, and exercises relevant to individuals suffering from anxiety disorders. Participants are provided free‐form text fields to document their work on assignments during and between modules. Therapists have access to all data submitted by participants on the online platform.

The treatment program was delivered through a secure online platform that complied with the European Union's General Data Protection Regulation (GDPR), employing encryption and mandatory two‐factor authentication for sign‐in. Therapists logged into the program weekly to review the participants´ work and provide feedback. Bidirectional text communication was available online, allowing participants to contact their therapist during treatment. Therapists spent approximately 15–20 min per patient per week. If participants had not logged in for a week, they received reminders via email or phone. The feedback provided in this study was similar to the approach used in Ivanova et al. (2016) study. The focus of therapist feedback was characterized by validating participants' progress, reinforcing positive behaviors, encouraging continued engagement, addressing problem‐solving, and supporting the application of therapeutic strategies to real‐life situations.

### 2.8 Therapists

All four therapists in this study were clinical psychology students in their final year of training. They had foundational knowledge of ACT and training in delivering CBT. To ensure the quality of assessment and written communication in the treatment, all therapists were trained in MINI 7.0.0, attended a two‐day course on the IACT treatment program, and received supervision from a licensed psychologist with expertise in internet‐based treatment.

### 2.9 Power Calculations

Limited research on the current treatment and population gave the study a partially exploratory character. It was estimated that a clinically meaningful outcome required at least a moderate effect, which could be detected with sufficient power by including 50 participants (Regional Ethical Review Board in Uppsala, Sweden, Dnr: 2018/451).

### 2.10 Data Analysis

Analyses were conducted using IBM SPSS Statistics version 28, R (v4.4.1), and SAS v9.4. Differences in demographic variables and dropout rates between the treatment and control groups were analyzed using independent *t*‐tests and *χ* ^2^ tests. Adherence to internet‐based treatments is measured through various methods, including the number of logins (Sieverink et al. 2017). This study assesses adherence based on the weekly completion of PDSS‐SR assessments.

The analyses focused on changes in symptoms of panic disorder (PDSS‐SR) and quality of life (BBQ) using an intention‐to‐treat approach, which included all participants initially randomized. The PDSS‐SR was administered at baseline and post‐treatment in both the treatment and control groups. Participants in the treatment group completed the PDSS‐SR weekly during the intervention (a total of 10 time points), primarily for the alliance‐related analyses. To ensure comparability with the control group, the outcome analyses included only the baseline and post‐treatment PDSS‐SR data. The BBQ was administered at two time points: pre‐treatment and post‐treatment in both groups. Multilevel modeling (MLM) was used for repeated measures to address dependencies in the data arising from multiple observations within the same individuals and to handle missing data effectively.

We used R packages lme4 v1.1–35 and lmerTest 3.1–3. To compute a longitudinal Linear Mixed Model (LMM) with an interaction effect. This method allowed us to assess the overall temporal changes in the treatment group while examining the interaction between the two study groups and time. Time was treated as a factor, with measurements taken at various time points, including pre‐ and post‐intervention and interim assessments within the treatment group. Our modeling approach included fixed effects for time, treatment condition, and interaction between time and treatment. Given the relatively limited number of available data points, we chose to model the time slope as a fixed effect.

We used two approaches to calculate effect sizes. To quantify the treatment effect over time, we computed the *d*_GMA‐raw_ effect size as proposed by Feingold (2019). This metric expresses the difference in estimated means between treatment and control groups at the final time point, standardized by the baseline standard deviation. The model was centered on the treatment group, with group comparisons based on the interaction effect.

This approach allows for interpretation in the same metric as Cohen's *d*, facilitating comparison with traditional repeated‐measures designs and across studies using growth modeling. Additionally, we also calculated between‐group effect sizes using the most reported method: the mean difference between groups divided by the pooled standard deviation of the raw data.

Following Cohen's (1988) guidelines, effect sizes were categorized as follows: a small effect size is considered above *d* = 0.20, a medium above *d* = 0.50, and a large above *d* = 0.80.

Fisher's exact test examined differences in post‐treatment diagnoses between the groups. The analysis was conducted using SAS v9.4. To be considered in remission, the participant had to be free from PD and agoraphobia diagnosis according to MINI. 7.0.0.

The alliance analysis included all four therapists and the participants in the treatment group. We used Pearson's correlation to investigate the relationship between participants' and therapists' assessment of the working alliance (measured by the WAI‐SR‐C and WAI‐SR‐T) and treatment outcomes (assessed using the PDSS‐SR pre‐ and post‐treatment). According to Cohen's (1988) guidelines, a correlation of 0.1 implies a weak relationship, 0.3 a medium relationship, and 0.5 a strong relationship. We used Cronbach's alpha to evaluate internal consistency.

We employed linear mixed models (LMM) to estimate between‐level and within‐level effects for the therapists and participants to analyze correlations during the ongoing treatment. Additionally, the analyses assessed overall changes within the treatment group. We also investigated whether the differences and changes in therapist and participant‐rated alliance were related to changes in PDSS‐SR. Time was treated as a variable, with weekly assessments centered at the midpoint of each five‐week period. Our analytical approach included fixed effects for time, grand mean centering to evaluate between‐person effects, person mean centering to evaluate within‐person effects, and random effects for both patient and time. We distinguished between‐person effects from weekly within‐person changes in alliance (variations around their mean) to isolate the process of alliance changes without the influence of between‐person differences. This distinction enabled us to determine whether increases or decreases in alliance are linked to changes in treatment outcomes or if participants with consistently higher alliance ratings, without fluctuations, achieve better overall treatment outcomes.

### 2.11 Language Support and Editing

During the final stages of the work, we used Grammarly and ChatGPT‐4, particularly for refining grammar and language clarity. The authors originally wrote the material, and the tool was used to refine it in its final phase. After using the tool, the authors reviewed and edited the content and take full responsibility for the material.

## 3 Results

### 3.1 Pre‐Treatment Assessment

The treatment group exhibited a significantly higher proportion of single participants (*χ* ^2^(2) = 8.267, *p* = 0.016). In contrast, the control group had a significantly greater proportion of participants who reported prior psychological treatment for the same/similar disorders (*χ* ^*2*^(1) = 5.148, *p* = 0.023). No significant differences were found between the groups regarding the remaining demographic variables or the pre‐treatment PDSS‐SR and BBQ measurements. Additionally, there were no significant differences between participants who dropped out and those who completed the final measurement. Before conducting formal statistical analyses, underlying distributional assumptions were examined, particularly skewness, kurtosis, outliers, and homogeneity of variance. Model assumptions were checked visually by plotting a histogram for every variable. No data transformations were deemed necessary after this inspection.

### 3.2 Adherence

58% (*n* = 27) of the treatment group participants completed PDSS‐SR assessments in all eight modules of the treatment program. Four participants finished more than half of the modules, while the remaining eight completed half or fewer. The average completion rate was five out of eight modules. Module four, which features interoceptive exposure, had a participation rate of 61%.

### 3.3 Primary Outcome

The intention‐to‐treat analysis conducted on the treatment group (*n* = 40) and control group (*n* = 39) showed a significant and negative effect of time (*p* < 0.001), indicating a substantial reduction in the primary outcome (PDSS‐SR) in the treatment group. Additionally, the interaction effect between the two conditions and time for the PDSS‐SR outcome measure was significant (*p* < 0.001), indicating a significantly better outcome in the treatment group. The fixed effects of Group, Time, and Group Time interaction explained 12.3% of the variance in PDSS‐SR (marginal R^2^), and the full model explained 63.7% of the variance in PDSS‐SR.

Both analyses reflect a large between‐group effect size. Based on a multilevel model (*n* = 79), the estimated effect size was *d*_GMA‐raw_ = 0.86, 95% CI [0.43, 1.29]. (See Table 3). For the observed values (*n* = 65) the estimated effect size was *d* = 0.92, 95% CI [0.40, 1.43]. For descriptive changes in PDSS‐SR and BBQ before and after treatment, see Table 4.

**TABLE 3:** Multilevel model (MLM) analysis of PDSS‐SR (panic symptom severity) and BBQ (quality of life) across time and group.

PDSS‐SR					BBQ			
Predictors	*b*	std. Beta	CI	*p*	*B*	std. Beta	CI	*p*
Intercept	16.61	−0.22	13.99 to 26.46	**< 0.001**	38.90	0.28	28.51 to 49.29	**0.001**
Group	−4.04	0.43	−7.69 to −0.39	**0.030**	−1.70	−0.52	−16.25 to 12.85	0.818
Time	−4.48	−0.43	−6.07 to −2.89	**< 0.001**	8.18	0.18	2.18 to 14.17	**0.027**
Group × Time	4.33	0.43	2.17 to 6.48	**< 0.001**	−6.76	−0.15	−14.87 to 1.35	0.101

*Note:* BBQ, Brunnsviken Brief Quality of Life Scale; PDSS‐SR, Panic Disorder Severity Scale—Self‐Rated. *p < 0.05.

**TABLE 4:** Changes in PDSS‐SR (panic symptom severity) and BBQ (quality of life) across time and group between Treatment Group and Waitlist Control.

Variable	Treatment group (*n* = 40)	Waitlist control (*n* = 39)
PDSS‐SR		
Pre mean (SD)	12.1 (5.1)	12.4 (5.0)
Post mean (SD)	8.40 (5.1)	12.3 (4.7)
Change (SD)	−3.73 (4.9)	−0.10 (3.7)
BBQ		
Pre mean (SD)	47.1 (22.8)	38.6 (20.2)
Post mean (SD)	53.5 (23.4)	39.7 (20.3)
Change	6.38 (14.6)	1.13 (16.5)

*Note:* BBQ, Brunnsviken Brief Quality of Life Scale; PDSS‐SR, Panic Disorder Severity Scale—Self‐Rated.

### 3.4 Exploratory Analysis

The analysis of the PDSS‐SR for participants with agoraphobia (*n* = 54) showed that the treatment group had a significant pre‐post improvement on the primary outcome measure (*p* < 0.001) as well as on group time interaction. Both analyses reflect a large between‐group effect size. Based on a multilevel model (*n* = 54), the estimated effect size was *d*GMA‐raw = 0.99, 95% CI [0.45,1.53] (see Table S5). Calculated on the observed values (*n* = 42) the effect size was *d =* 1.22, 95% CI [0.59,1.86]. (see Table S5). Both results indicate a large effect in the treatment group relative to the control group.

### 3.5 Secondary Outcome

The analysis indicated a significant and positive effect of time, demonstrating an improvement of the secondary outcome (BBQ), in the treatment group (*p* = 0.027). However, the analysis showed no significant interaction effect between time and group (*p* = 0.101); hence, the improvement over time was not more substantial in the treatment condition compared to the control condition (see Table 5).

**TABLE 5:** Descriptive data for therapist‐ and client‐rated working alliance.

Week	Measurement	*N*	*M*	SD
1	WAI‐SR‐C	34	39.24	11.64
	WAI‐SR‐T	39	27.26	5.28
2	WAI‐SR‐C	31	40.52	12.22
	WAI‐SR‐T	39	31.24	8.81
3	WAI‐SR‐C	28	45.14	11.16
	WAI‐SR‐T	39	31.33	8.8
4	WAI‐SR‐C	23	45.52	11.87
	WAI‐SR‐T	39	31.59	9.65
5	WAI‐SR‐C	21	46.43	11.54
	WAI‐SR‐T	39	32.15	11.11
6	WAI‐SR‐C	24	46.67	11.81
	WAI‐SR‐T	39	33.28	13.27
7	WAI‐SR‐C	19	47.52	12.92
	WAI‐SR‐T	39	33.26	13.7
8	WAI‐SR‐C	19	49.26	10.46
	WAI‐SR‐T	39	32.33	12.58
9	WAI‐SR‐C	17	50.71	10.15
	WAI‐SR‐T	39	32.21	13.2
10	WAI‐SR‐C	27	46.78	12.92
	WAI‐SR‐T	39	32.62	13.34

*Note:* WAI‐SR‐C, Working Alliance Inventory—Short Revised, Client Version; WAI‐SR‐T, Working Alliance Inventory—Short Revised, Therapist Version.

### 3.6 Remission

At the post‐treatment MINI interview, 42.9% (12 of 28) of the participants in the treatment group and 5.7% (2 of 35) of the participants in the control group no longer met the criteria for PD. This showed a significant difference between groups (*p* < 0.001).

After treatment, 27.8% (5 out of 18) of participants in the treatment group and 4.3% (1 out of 23) of participants in the control group with agoraphobia no longer met the criteria for PD. This showed a significant difference (*p* = 0.037). Regarding the diagnosis of agoraphobia, 44.4% (8 of 18 participants) in the treatment group and 17.4% (4 of 23 participants) in the control group no longer meet the diagnostic criteria. This result did not show a significant difference (*p* = 0.062).

### 3.7 Working Alliance

The number of participants reporting alliance assessments varied across the weeks, ranging from 17 to 34 each week. One participant who only took part in the initial and final assessments was excluded to maintain consistency in the dataset. Therapists conducted all the assessments, and data on 39 weekly therapeutic alliances were recorded. Internal consistency was high (*α* = 0.94–0.98) across all assessments.

The correlations between alliance ratings from Weeks 1–10 and the final PDSS‐SR treatment outcome ranged from 0.06 to 0.28 for client‐reported alliance (WAI‐SR‐C) and from 0.02 to 0.32 for the therapist‐reported alliance (WAI‐SR‐T) with no statistical significance (*p* > 0.05; see Table S3). The mean WAI‐SR‐C score increased from 39.2 in week one to a peak of 50.71 in week nine and dropped to 46.8 post‐measurement. The WAI‐SR‐T mean score was 27.3 in Week 1, reaching 32.6 at post‐measurement, showing a more consistent trend. The descriptive data for therapist‐ and client‐rated working alliance are presented in Table 5. The variance in average scores results from the difference in the number of questions between WAI‐SR‐T and WAI‐SR‐C, not a differing valuation of the alliance itself. The initial correlation between participant and therapist measurement correlation was non‐existent. However, strong correlations (0.41–0.71) were observed from week three onwards (see Table S4).

We evaluated the weekly relationship between alliance ratings (WAI‐SR‐C and WAI‐SR‐T) and treatment outcomes (PDSS‐SR) throughout the treatment period within the treatment group. In this model, time was centered at week five. The predictors WAI‐SR‐C and WAI‐SR‐T were grand mean centered on evaluating between‐person effects and person mean centered on examining within‐person effects. The grand mean centered WAI‐SR‐C and WAI‐SR‐T were insignificant, indicating that participants with an overall higher patient (WAI‐SR‐C) or therapist‐rated (WAI‐SR‐T) alliances do not have better outcomes. More importantly, looking at the within‐person effect (change) between alliance assessments during weeks 1–10 and PDSS‐SR, we found that while changes in WAI‐SR‐C were not significantly related to the outcome, changes in WAI‐SR‐T were significantly associated with the outcome. This indicate that increases in within centered WAI‐SR‐T were linked to reductions in PDSS‐SR (see Table 6).

**TABLE 6:** Multilevel Model (MLM) Analysis of PDSS‐SR (Panic Symptom Severity) and WAI‐SR (Working Alliance Inventory, Client and Therapist Versions) Across Time and Group.

Predictors	*b*	CI	*p*
Intercept	9.71	8.43 to 10.98	**< 0.001**
WAI‐SR ‐T Between person	0.13	−0.04 to 0.29	0.139
WAI‐SR ‐C Between person	−0.08	−0.20 to 0.04	0.201
WAI‐SR ‐T Within person	−0.13	−0.21 to −0.05	**0.001**
WAI‐SR ‐C Within person	0.04	−0.07 to 0.16	0.464
Time in treatment (week)	−0.47	−0.69 to −0.24	**< 0.001**
Random effects			
σ^2^	7.98		
τ_00 ID_	10.33		
τ_11 ID.week_	0.20		
ρ_01 ID_	0.13		
ICC	0.60		
*N*_ID_	35		
Observations	245		
Marginal *R* ^2^/conditional *R* ^2^	0.165/0.668		

*Note:* MLM, multilevel model.; PDSS‐SR, Panic Disorder Severity Scale—Self‐Report; WAI‐SR‐C, Working Alliance Inventory—Short Revised, Client Version; WAI‐SR‐T, Working Alliance Inventory—Short Revised, Therapist Version. *p < 0.05.

## 4 Discussion

The primary aim of this study was to evaluate the efficacy of an internet‐based Acceptance and Commitment Therapy (IACT) program, modified to include interoceptive exposure, for treating PD with or without concurrent agoraphobia. The study used an RCT and a systematic assessment using valid psychometric instruments. In addition, a clinical evaluation, including the semi‐structured interview MINI 7.0.0, was conducted to gather insights beyond mere symptom reduction. Weekly PDSS‐SR measurements allowed continual data collection from participants who discontinued treatment. The participants' educational backgrounds closely matched Sweden's average, thereby enhancing generalisability (Statistikmyndigheten 2022).

The modified IACT treatment showed a strong effect in reducing panic disorder symptoms. The effect size calculated from observed values was Cohen's d = 0.96, while the model‐predicted effect size based on the multilevel model was *d*_GMA‐raw_ = 0.86, (Feingold 2019). There are few studies on ACT and IACT that focus on PD and provide comparable control conditions. Thus, this study's result exceeded Han and Kim's (2022) meta‐analytic average effect of IACT for anxiety disorders (SMD = 0.39) using non‐active control conditions. However, our results are lower than the effect size for CBT for PD reported in a systematic review and meta‐analysis (*g* = 1.22), where the control conditions were waitlist and information‐based controls (Stech et al. 2020).

Based on assessments using the MINI interview, 43% of participants in our study were determined to be in remission from PD, meaning they no longer met the diagnostic criteria, compared to 6% in the control group. Using the meta‐analysis by Springer et al. (2018) as a comparison, the remission rate in the treatment group is somewhat below the average remission rate for face‐to‐face CBT for PD (mean = 55.5%). However, few studies included in that meta‐analysis used a formal diagnosis as a criterion for remission (Springer et al. 2018). Clinician‐rated remission is by certain researchers regarded as a conservative method, and recommended over self‐assessment (Cuijpers et al. 2010). However, others have discussed the pros and cons of various remission measures (Springer et al. 2018).

An earlier RCT, that tested the same IACT program before it included exposure elements, found no significant effects concerning PD (Ivanova et al. 2016). However, that study had few PD participants, and the within‐group effects were large (*d* = 1.18 for the guided group and *d* = 0.99 for the unguided group). Anxiety Help integrated interoceptive exposure to target panic symptoms and enhance patient‐recognition within a transdiagnostic framework (Craske and Barlow 2014; Ivanova et al. 2016). However, due to our study's design, including the inability to determine who had read or completed the interoceptive exposure exercises in module 4, definitive conclusions regarding the significance of this modification cannot be drawn.

While integrating interoceptive exposure into IACT treatment provides a comprehensive approach to PD treatment, challenges can arise when combining these methods. One challenge lies in balancing the acceptance‐oriented framework of ACT with the more change‐oriented techniques of CBT. ACT encourages patients to accept their anxiety and panic symptoms and at the same time, CBT focuses on modifying cognitive distortions and avoidance behaviors associated with panic disorder. ACT's emphasis on mindfulness and acceptance exercises can be problematic to reconcile with CBT's more directive and problem‐solving strategies. The differences between these perspectives can confuse patients who might struggle to resolve the contradiction between accepting symptoms and trying to modify them. Ensuring that patients understand the complementary nature of these approaches and how to combine them is essential. Integrating ACT and CBT requires consideration of the sequencing and timing of interventions. In practice, this integration requires a thorough understanding of both methodologies, emphasizing the delivery of interventions coherently and systematically to avoid confusing the patient. Despite the challenges of integrating these methods, the approach holds promise when executed with care and precision.

Previous studies indicate that agoraphobia often worsens psychiatric conditions (Grant et al. 2006; Inoue et al. 2016). In our study, participants with agoraphobia scored higher on PDSS‐SR at both pre‐ and post‐measurement. However, those in the treatment group showed significant improvement in PD symptoms, with an even larger effect size than in the overall sample. While PD symptoms were substantially reduced, remission rates for participants with concurrent agoraphobia were lower. Furthermore, the effect on agoraphobia remission did not reach statistical significance. This suggests that while the intervention had a positive effect on panic disorder symptoms, its impact on diagnostic remission was less pronounced.

We used the Brunnsviken Brief Quality of Life Scale (BBQ) as a secondary outcome. While overall quality of life improved among participants in the treatment group, our analysis showed no significant differences between groups. This is consistent with meta‐analyses on both IACT (Han and Kim 2022) and ICBT (Maj et al. 2023), which demonstrate no or minor effects regarding the effect on quality of life. Regardless of whether the treatment focuses on symptoms or quality of life, more gradual improvements in quality of life seem likely. A longer follow‐up may provide a better assessment of changes in this outcome.

Additionally, the study had a secondary aim to examine whether therapist‐ and client‐rated working alliance was related to treatment outcomes. In this study, we did not find a significant correlation between single‐week measures of working alliance and treatment outcome. However, changes in WAI‐SR‐T scores during treatment were significantly associated with outcome. This indicates that an increasing therapist‐assessed alliance had a significant positive correlation with improvements in the primary measure. Therapist‐rated alliance may provide valuable insights into predicting outcomes in internet‐based treatments and deserves further exploration. Unlike patients, who assess both the online material and therapeutic interactions, therapists focus solely on the patient's activity. Therapists may be more adept at detecting the level of the patient's engagement, which can influence how the therapist evaluates their alliance. This might correspond with Bendelin et al.'s (2011) suggestion that patients actively participating in internet‐based treatment and exercises tend to achieve better outcomes. Our findings also highlight the importance of monitoring the working alliance as a process rather than relying on isolated measurements.

### 4.1 Limitations

Certain limitations must be considered regarding this study. While employing a randomized controlled trial (RCT) with a waitlist is common practice, it introduces specific biases that may have exaggerated a positive outcome. Furthermore, convenience sampling could have affected the results and their generalisability, as illustrated by the ratio of women (5:1). Additionally, variations in marital status and prior treatment suggest possible allocation bias. Attrition bias may also have impacted the analysis of remission, especially if dropouts differed significantly from those who completed the study. Nevertheless, there were no significant baseline differences in PDSS‐SR or BBQ between groups, including those who withdrew from the study.

The lack of blinding may have affected the therapist's assessment in the MINI interview. However, using PDSS‐SR and the intention‐to‐treat approach helped reduce these potential biases. Including 14 participants whose pre‐treatment PDSS‐SR scores were below the initial inclusion criteria may have reduced the perceived treatment efficacy and generalisability of the results. Still, the average baseline score was within the moderate range for panic disorder symptoms (Furukawa et al. 2009).

A longer follow‐up, especially for BBQ, would have been beneficial, given that ACT focuses on valued living beyond symptom remission. This is especially relevant as one‐third of patients with panic disorder remain challenging to treat (Freire et al. 2016), and that ACT shows potential for those unresponsive to CBT (Gloster et al. 2015). Inconsistent client participation in assessing the working alliance and a dropout rate exceeding 30% limit the conclusions we can draw about the relationship between the alliance and outcomes. Another limitation is that, we did not carry out further analyses of the link between working alliance and outcome, including lagged relationships. As a result, we cannot infer causality.

Additionally, we lack data on the extent of contact between participants and therapists. Nevertheless, as with most internet‐based treatments, the digital material provided most of the treatment content in this study, while the therapists' role was to support participants' engagement with this material. Additionally, completing PDSS‐SR assessments does not fully capture participants' overall engagement with the treatment and its components. Future research should explore alternative approaches to examining the working alliance, considering both the therapeutic material and the therapist. Additionally, it should incorporate a broader range of adherence indicators that integrate both quantitative and qualitative aspects.

### 4.2 Clinical Implications and Suggestions for Future Research

This study evaluated the efficacy of an IACT program incorporating interoceptive exposure, based on the idea that combining ACT with these CBT components enhances both effectiveness and applicability.

ACT's approach is consistent with the broader concept of transdiagnostic treatment, which aims to address common underlying processes across various disorders, making it potentially more generalisable compared to disorder‐specific approaches (Cuijpers et al. 2023; Dalgleish et al. 2020). However, diagnosis‐specific treatments like CBT target well‐defined symptoms and may provide greater clarity, enhance understanding, and thereby support adherence to the treatment. Research has also emphasized the importance of interoceptive exposure in relation to PD (Craske and Barlow 2014).

While this study suggests that IACT can be effective for treating panic disorder (PD), it does not investigate the potential advantages or disadvantages of incorporating interoceptive exposure into an IACT treatment. Studies comparing “ordinary” IACT, exposure‐modified IACT, and ICBT could provide valuable insights into the efficacy of these treatments. This could also give more insight into patients' experiences of the perceived compatibility of different components, their prioritization between various forms of exposure strategies, and the overall coherence of the treatment. This is also interesting, as it remains unclear whether substituting specific material significantly impacts treatment outcomes (Cuijpers et al. 2019).

Whether blending different treatment rationales creates confusion among participants is essential, as providing a clear treatment rationale is associated with improved outcomes (Ahmed and Westra 2009; Olofsdotter Lauri et al. 2022). Complex and unclear materials can diminish the effectiveness and usability of internet‐based programs (Johansson et al. 2015). The lack of nonverbal cues and spontaneous feedback in internet‐based treatments highlights the essential role of explicit, well‐structured content in promoting engagement and perhaps also has an impact on working alliance.

## 5 Conclusion

This study aimed to evaluate the effectiveness of a modified IACT treatment that incorporates interoceptive exposure for panic disorder symptoms. The results demonstrated a significant symptom reduction, supporting the efficacy of IACT for individuals with PD, including those with comorbid agoraphobia. The study contributes to the growing body of knowledge on working alliance in internet‐based treatments, showing that increases in the therapist‐rated alliance were positively correlated with treatment outcome, highlighting its potential as a predictive factor. Integrating interoceptive exposure within an IACT framework highlights the possibility of combining transdiagnostic and diagnosis‐specific approaches. However, further research is needed to evaluate its comparative efficacy and coherence in treating PD.

## Author Contributions

All authors made substantial contributions to the study, and L.B., as first author, took primary responsibility for the conceptual framing, interpretation of findings, and manuscript drafting. T.H. and K.V., with significant input from E.E., I.H., N.J., and N.P., conceptualized and designed the study. E.E., I.H., N.J., and N.P. carried out the investigation, with central contributions from T.H., K.V., and E.R.; P.B., L.B., and T.H. made the major methodological contribution, with additional substantial input from K.V., L.B., and S.W. during the study process. L.B., S.W., and P.B. conducted the formal analyses, with additional input from K.V. and T.H.; L.B. and S.W. were responsible for funding acquisition. L.B. wrote the first draft of the manuscript, and S.W., K.V., T.H., and P.B. contributed with crucial input as well as with review and editing of all parts of the manuscript. E.E., E.R., I.H., N.J., and N.P. reviewed the manuscript. All authors revised the manuscript critically for important intellectual content, approved the final version to be published, and agreed to be accountable for all aspects of the work.

## Disclosure

Permission to reproduce: Figures from the treatment content (Psykologpartners).

## Ethics Statement

This RCT was approved by the Regional Ethical Review Board in Uppsala, Sweden (Dnr: 2018/451). All participants provided written consent for their involvement, fully aware that the findings would be published in a scientific journal. Participants were informed that the study results would be presented in an aggregated format at the group level, ensuring the confidentiality of individual identities.

## Conflicts of Interest

During the data collection, Kristofer Vernmark and Ella Radvogin were employed by Psykologpartners, the company that developed and distributed the treatment program evaluated in this study. The other authors have no conflicts of interest.

## Supporting information


**Figure S1:** Visual examples on treatment content.

**Table S1:** Content of the Module 4.
**Table S2:** Information about the treatment program Anxiety Help.
**Table S3:** Correlations between client and therapist alliance measurements and the outcome, Week 1–10.
**Table S4:** Correlation between client (WAI‐C SR) and therapist (WAI‐T SR Week 1–10).
**Table S5:** MLM analysis of PDSS‐SR for participants with agoraphobia.

## Data Availability

The data that support the findings of this study are available from the corresponding author upon reasonable request.
