# Supplementary material for: Internet‐Based Acceptance and Commitment Therapy With Interoceptive Exposure for Panic Disorder: A Randomized Controlled Trial and Working Alliance Analysis
Source: Scand J Psychol. 2025 Nov 28;67(2):605–19. doi: 10.1111/sjop.70045 (PMC12984000; doi:10.1111/sjop.70045)

Supplementary materials (Figures**)**

Internet-Based Acceptance and Commitment Therapy with Interoceptive Exposure for Panic Disorder: A Randomised Controlled Trial and Working Alliance Analysis

**Supplement Figure 1.** *Visual examples on treatment content*

**Supplement Figure 1**

*Visual examples on treatment content*


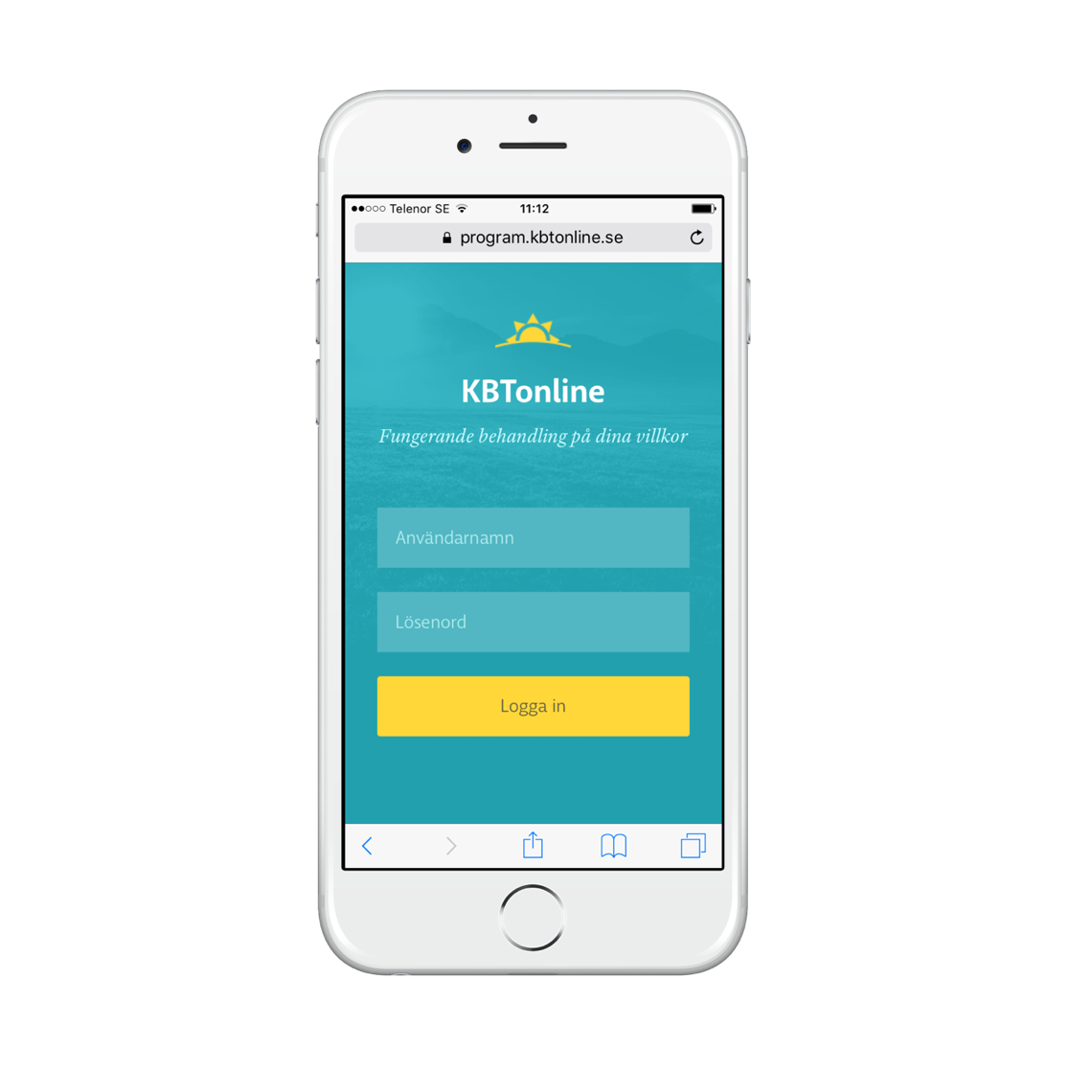


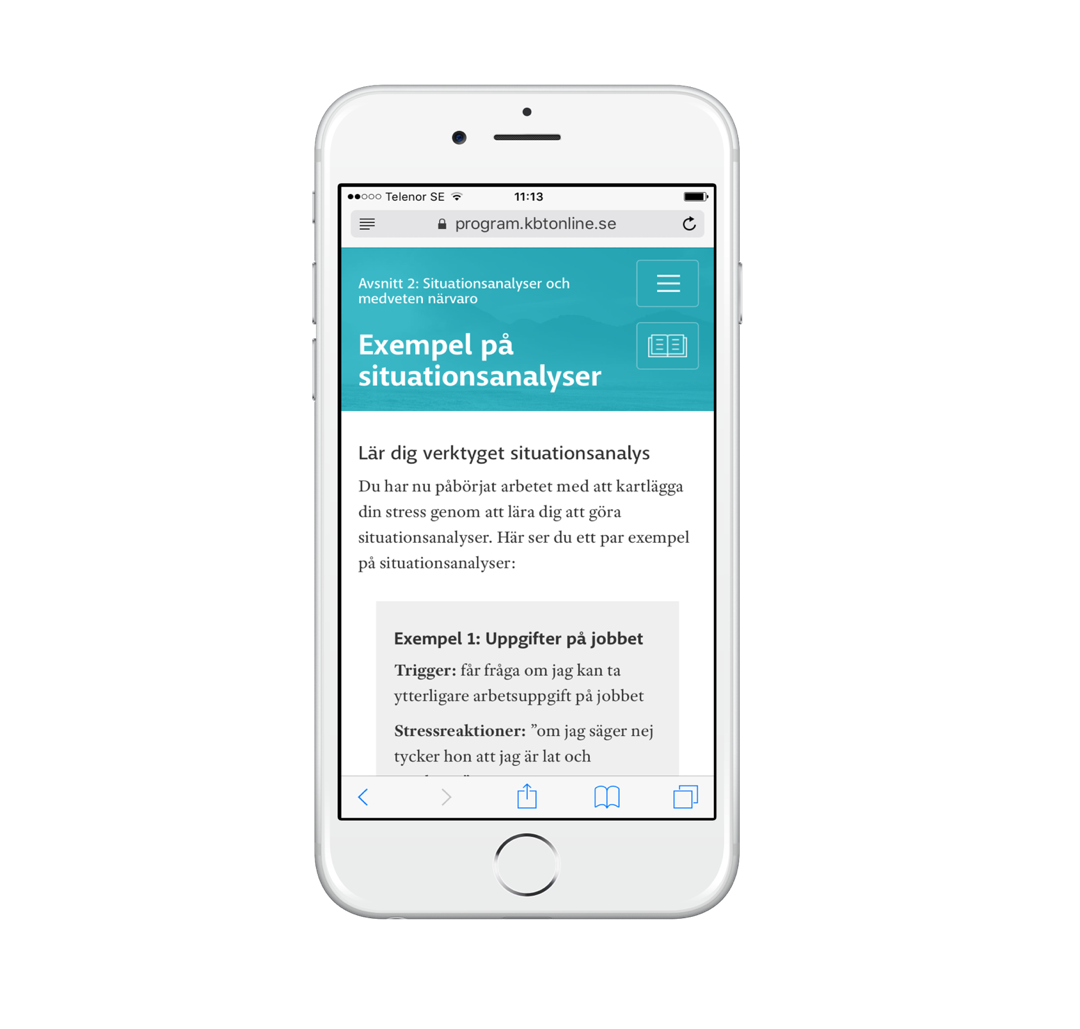


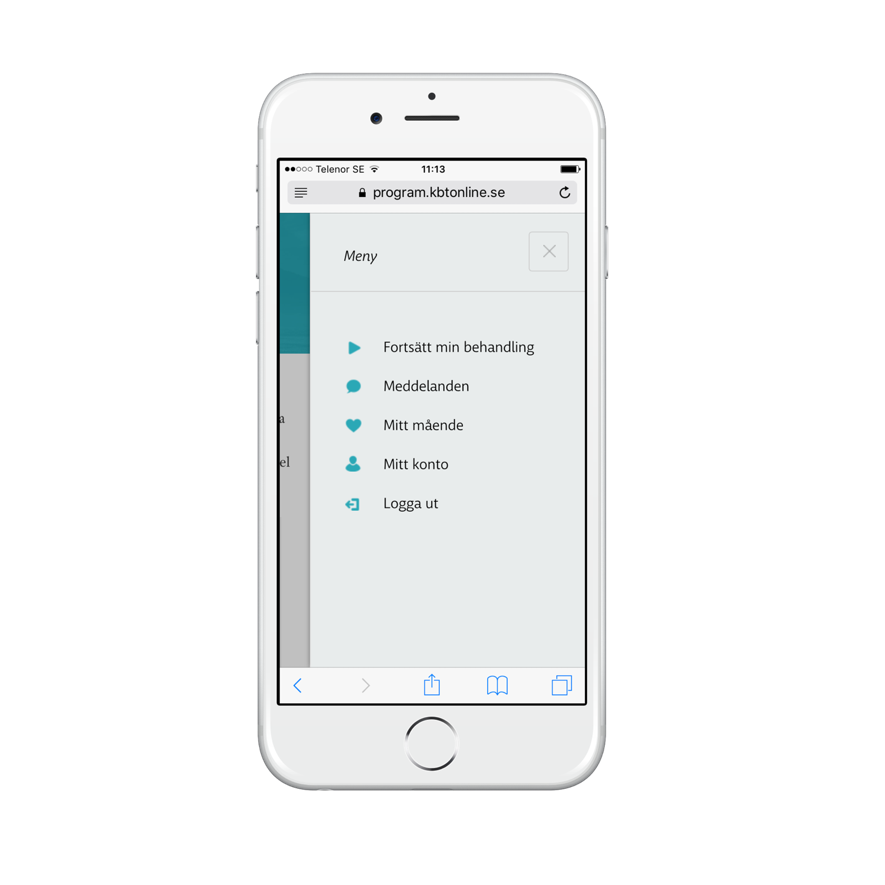


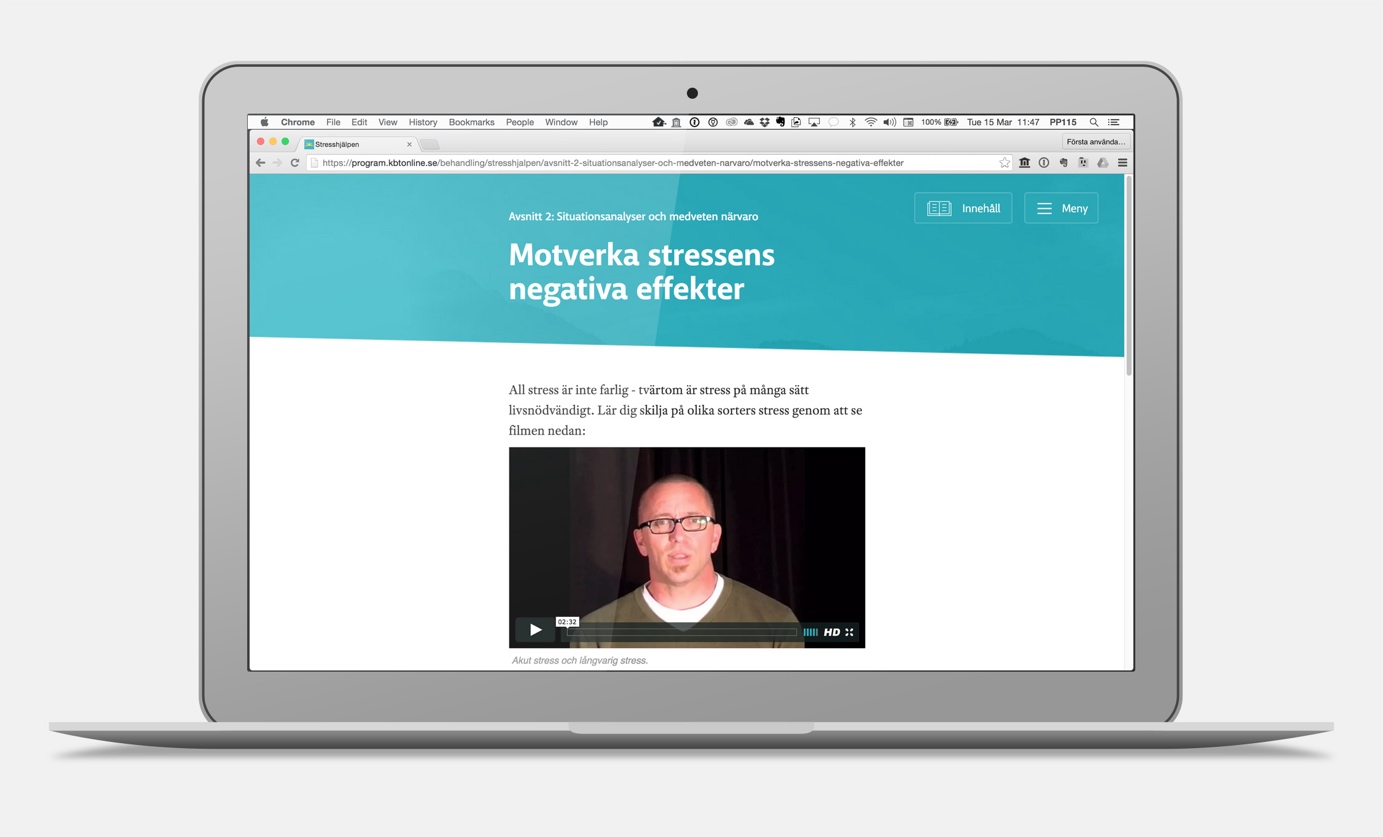


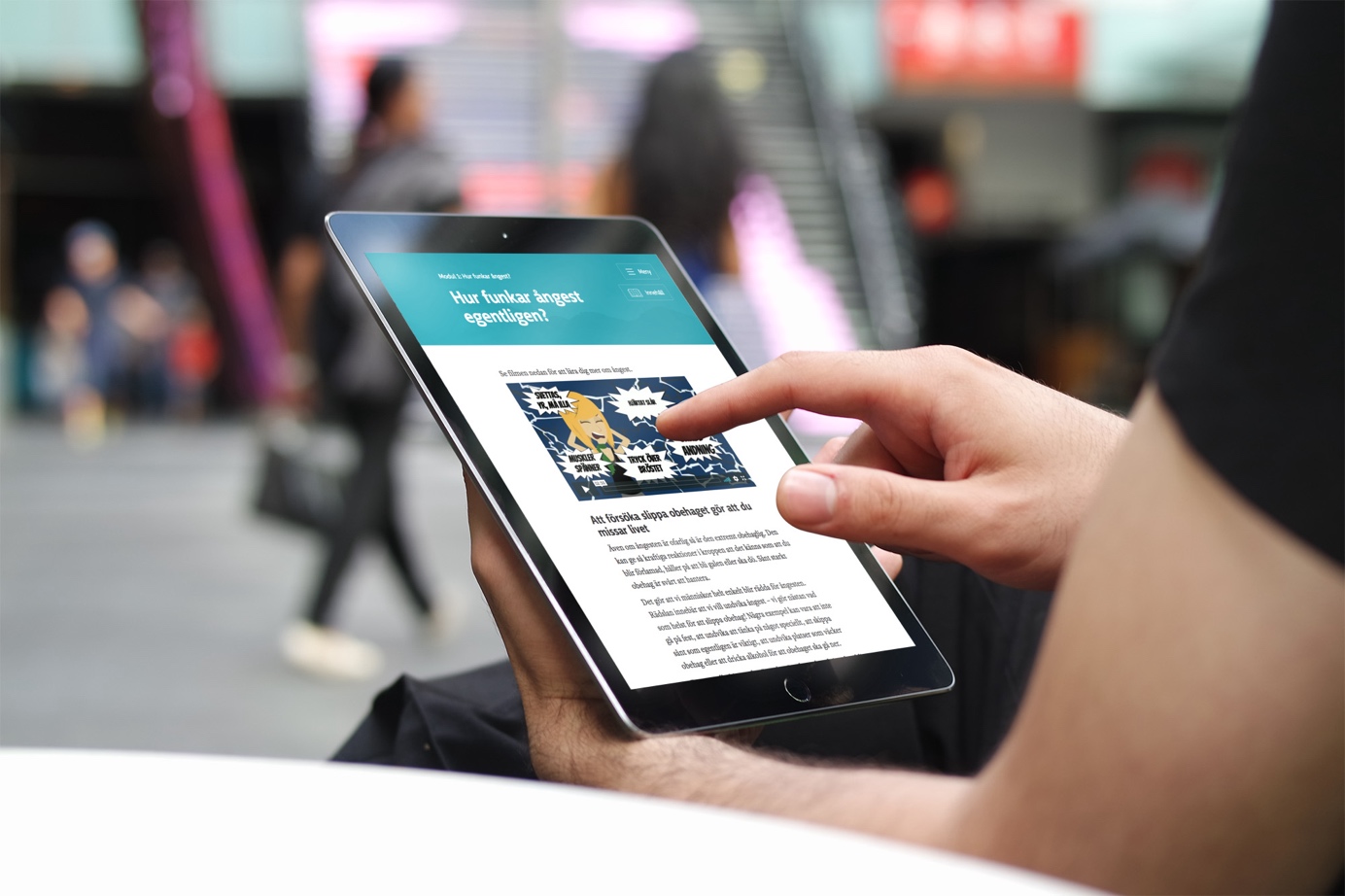

Supplement: Supplementary file 1 — Figure S1: Visual examples on treatment content. [file SJOP-67-605-s001.docx]
