# Supplementary material for: Internet‐Based Acceptance and Commitment Therapy With Interoceptive Exposure for Panic Disorder: A Randomized Controlled Trial and Working Alliance Analysis
Source: Scand J Psychol. 2025 Nov 28;67(2):605–19. doi: 10.1111/sjop.70045 (PMC12984000; doi:10.1111/sjop.70045)
Supplement: Supplementary file 2 — Table S1: Content of the Module 4. Table S2: Information about the treatment program Anxiety Help. Table S3: Correlations between client and therapist alliance measurements and the outcome, Week 1–10. Table S4: Correlation between client (WAI‐C SR) and therapist (WAI‐T SR Week 1–10). Table S5: MLM analysis of PDSS‐SR for participants with agoraphobia. [file SJOP-67-605-s002.docx]

Supplementary materials (Tables)

Internet-Based Acceptance and Commitment Therapy with Interoceptive Exposure for Panic Disorder: A Randomised Controlled Trial and Working Alliance Analysis

# List of Tables

**Supplementary Table 1.** *Content of the Module 4*

**Supplementary Table 2**. *Information about the treatment program Anxiety Help*

**Supplementary Table 3**. *Correlations between client and therapist alliance measurements and the outcome, week 1–10.*

**Supplementary Table 4**. *Correlation between client (WAI-C SR) and therapist (WAI-T SR week 1–10.*

**Supplementary Table 5.** *MLM analysis of PDSS-SR for participants with agoraphobia*

**Supplementary Table 1.**

*Content of the Module 4*

| **Brief summary of key points in Module 4**.  These themes highlight the essential role of acceptance in managing emotions and making effective life choices.  *Acceptance*: The phrase, “Grant me the strength to change what I can, the serenity to accept what I cannot change, and the wisdom to know the difference,” encapsulates the essence of acceptance. It involves the capacity to observe and understand what we can and cannot influence.    *Daring to Feel*: Acceptance also necessitates embracing and fully engaging with difficult memories, thoughts, and emotions, instead of attempting to suppress or evade them.  The goal of acceptance is not to feel better—but to become better at feeling.    *Focus on What Matters*: Exposure functions similarly. The better prepared you are to confront your anxiety in every situation you face, the less likely it is that anxiety will significantly affect your life. Engaging in anxiety-provoking situations while focusing on effective behaviours rather than avoidance strategies is a skill you will continuously refine in the Anxiety Help program.    *What Can You Change?* Trying to control or eliminate unwanted thoughts and feelings often makes them more persistent. Acceptance teaches us to recognise this futility and instead allows us to let these internal experiences exist without attempting to suppress them.    **A summary of the text on exposure research**  Exposure therapy is effective in reducing anxiety by teaching the brain that anxiety is not harmful. By repeatedly facing anxiety in various forms, such as through physical activities that elevate your heart rate or cause dizziness, your brain learns that these sensations are manageable. Over time, this practice helps shift the belief that discomfort must be avoided towards the understanding that anxiety can be tolerated.    To maximise learning, it is essential to vary your exposure techniques and gradually reduce reliance on safety behaviours—actions taken to alleviate immediate discomfort. By doing so, you enable your brain to fully process and adapt to anxiety-inducing situations, thereby reducing both fear and anxiety over time. The new understanding-“anxiety is unpleasant, but I can manage it”-begins to supplant the old belief-“I must eliminate my discomfort." Consequently, you’ll feel freer to focus on what truly matters in your life.    Creating specific exposure exercises tailored to your fears-such as inducing shortness of breath, palpitations, or dizziness—can help you confront and overcome these sensations, leading to greater freedom and a focus on what truly matters in life. Afterwards, reflect on whether the experience matched your catastrophic thoughts and how reality differed from your fears.    *Daring to Know What Your Monsters Feel like:* There is an alternative approach to confronting your anxiety that is known to produce positive outcomes and can, over time, alleviate the uncomfortable feelings in your environment. Confront anxiety by actively triggering the physical sensations that evoke fear. By doing this, you can gain new experiences and realise that these sensations may not be as dangerous as you once believed. The aim of this experiment is to identify the bodily sensation you fear, provoke this sensation, allow the experience to unfold naturally, and remain with it until it subsides without resorting to safety behaviours.  **Main Exercise Module 4**  This week, practice facing your discomfort with acceptance and willingness instead of avoidance. The aim is to engage in activities you value while allowing any discomfort (your "monsters") to be present. For example, if you have a panic disorder, you might go for a run, experience palpitations, yet still complete the run.    *Reflect:* Did the experience match your catastrophic thoughts? How did reality differ? |
| --- |

**Supplementary Table 2**.

*Information about the treatment program Anxiety Help*

| Anxiety Help was designed by the private psychology company Psykologpartners in 2011 and is commercially available. The intervention is described as a transdiagnostic treatment for anxiety problems in adults from 18 years of age, specifically targeting the diagnoses of social phobia, panic disorder, agoraphobia, generalised anxiety disorder, obsessive-compulsive disorder, post-traumatic stress disorder and anxiety disorder without further specification (UNS). The program is to be administered as guided internet treatment where therapists must have at least basic CBT training and at least 2 days of training in internet treatment and in the program itself. The program is based on Acceptance and Commitment therapy (ACT).    Total number of treatment modules in the program is 8 and the recommended treatment time is 10 weeks. The treatment includes assignments/exercises and the recommended feedback from the internet therapist to the patient is once a week. Assessments are included in the program and include Anxiety, Quality of Life and Values (Directed Action).    *Number of words*: ca. 17,500  *Video*: ca. 20 min  *Audio exercises*: ca. 30 min |
| --- |

**Supplementary Table 3**

*Correlations between client and therapist alliance measurements and the final treatment outcome, week 1–10.*

| Week | 1 | 2 | 3 | 4 | 5 | 6 | 7 | 8 | 9 | 10 |
| --- | --- | --- | --- | --- | --- | --- | --- | --- | --- | --- |
| PDSS-SR  WAI-C SR | .05 | .28 | .18 | .20 | .08 | .20 | .13 | - .13 | -.03 | .20 |
| PDSS-SR  WAI-T SR | -.32 | - .10 | .15 | .15 | .09 | .13 | .07 | .02 | - .06 | - .04 |

*WAI-C=Working Alliance Inventory-Client PDSS-SR=Panic Disorder Severity Scale-Self Rated*

*WAI-T=Working Alliance Inventory-Therapist* *PDSS-SR=Panic Disorder Severity Scale-Self*

**Supplementary Table 4**

*Correlation between client (WAI-C SR) and therapist (WAI-T SR) week 1–10.*

| Week | 1 | 2 | 3 | 4 | 5 | 6 | 7 | 8 | 9 | 10 |
| --- | --- | --- | --- | --- | --- | --- | --- | --- | --- | --- |
|  | r=.265 | r=.413 | r=.569* | r=.549* | r=.685* | r=.652* | r=.710* | r=.638* | r=.695* | r=.613* |

*WAI-C=Working Alliance Inventory-Client WAI-T=Working Alliance Inventory-Therapist. *Significant*

**Supplementary Table 5**

*MLM analysis of PDSS-SR for participants with agoraphobia*

|  | **PDSS-SR** | | | | | |
| --- | --- | --- | --- | --- | --- | --- |
| *Predictors* | *Estimates* | *std. Beta* | *CI* | *standardized CI* | *p* | *std. p* |
| (Intercept) | 0.96 | -0.28 | 0.32 – 1.60 | -0.61 – 0.05 | **0.004** | 0.091 |
| grupp f [2] | -0.94 | 0.47 | -1.82 – -0.05 | 0.01 – 0.93 | **0.040** | **0.043** |
| tid | -0.98 | -0.48 | -1.39 – -0.58 | -0.68 – -0.28 | **<0.001** | **<0.001** |
| grupp f [2] × tid | 0.99 | 0.48 | 0.45 – 1.53 | 0.22 – 0.75 | **<0.001** | **<0.001** |
| **Random Effects** | | | | | | |
| σ^2^ | 0.40 | | | | | |
| τ_00_ _kod_ | 0.51 | | | | | |
| ICC | 0.56 | | | | | |
| N _kod_ | 54 | | | | | |
| Observations | 96 | | | | | |
| Marginal R^2^ / Conditional R^2^ | 0.150 / 0.625 | | | | | |
